# Predictive Factors of Major Lower Extremity Amputations in Diabetic Foot Infections: A Cross-sectional Study at District Hospital in Malaysia

**DOI:** 10.5704/MOJ.1911.008

**Published:** 2019-11

**Authors:** RY Kow, CL Low, JK Ruben, MZ Zaharul-Azri, BC Lim

**Affiliations:** Department of Orthopaedics, Hospital Tengku Ampuan Afzan (HTAA), Kuantan, Malaysia.; * Department of Radiology, Hospital Tengku Ampuan Afzan, Kuantan, Malaysia; ** Department of Orthopaedic, Hospital Kuala Lipis, Kuala Lipis, Malaysia

**Keywords:** diabetes, foot, ulcer, infection, amputation

## Abstract

**Introduction:** Diabetic foot infection, a complication which can lead to lower limb amputation, is a major source of morbidity and mortality in Malaysia. The objective of this study was to determine the predictive factors of major lower limb amputation among patients with diabetes mellitus in a cluster of three district hospitals in Pahang, Malaysia.

**Materials and Methods:** This cross-sectional study involved 170 patients who had undergone surgical interventions for diabetic foot infections at three district hospitals from 1st of September 2014 to 31st December 2015. The predictors for major amputation of lower limb were determined using simple logistic regression (LR) and forward LR multiple logistic regression.

**Results:** A total of 21 patients had undergone major amputations of lower limb (15 transtibial and 6 transfemoral). The following factors were associated with major amputation of lower limb; longer duration of disease, age ≥ 60 years, patients from Bentong Hospital, presence of hypertension, presence of fever, history of multiple limb-salvaging surgeries, monomicrobial culture, necrotising fasciitis, anemia and leukocytosis. Upon forward LR multiple logistic regression, only duration of disease, history of more than three previous limb-salvaging surgeries and total white blood cell count ≥15X109/L were found to be significant as predictive factors of major amputation of lower limb.

**Conclusion:** Among the factors analysed in this study, a longer duration of disease, raised total white blood cell count and history of more than three limb-salvaging surgeries were identified as predictors for major amputation of lower limb in diabetic foot infections using stepwise logistic regression analysis.

## Introduction

Diabetes mellitus is a major non-communicable disease in Malaysia. With a prevalence of 17.5% in 2015, this figure represents an increase of more than 50% from the previously reported 11.6% in 2006^1, 2^. Diabetes mellitus is associated with a variety of macro- and microvascular-related complications^3^. One of the major complications is diabetic foot ulcer^3^. It is reported that up to 15% of the diabetic patients developed diabetic foot ulcers over the course of their diseases^4^. Diabetic foot infection, a complication resulting from an interplay of ischemia, ulceration and infection, poses a major source of morbidity and it has accounted for substantial health care cost and resources^5^.

Amputations, either minor or major, are important surgical procedures with the purpose to remove the diseased tissue and to optimise ambulatory rehabilitation in patients with diabetic foot infections^6, 7^. Major amputation of lower limb, defined as surgery performed above the ankle, is often associated with post-operative morbidity and mortality^8, 9^. Major amputation of lower extremity may also render the patient immobile, resulting in severe economic, social and psychological effects on the patient and his family^10^. A population-wide study by Yusof *et al* in Malaysia shows that major amputations account for more than 59% of total amputations^10^. Various risk factors of amputation have been identified in diabetic patients in Malaysia, but none of the researches focused on the rural population^7, 11-13^.

This study aimed to evaluate the predictive factors of major lower limb amputation among patients with diabetes mellitus at a cluster of three district hospitals in Malaysia. This is especially important in rural areas where all high risk patients presenting to the district hospitals can be identified and early referrals to the tertiary hospitals can be made to prevent devastating outcomes.

## Materials and Methods

This was a cross-sectional study involving patients from a cluster of three district hospitals in Malaysia. With Kuala Lipis Hospital serving as a referral center, patients with complicated diabetic foot infections were referred to Kuala Lipis Hospital from other district hospitals and health clinics. All patients who had undergone surgical interventions for diabetic foot infections from 1st September 2014 to 31st December 2015 in Kuala Lipis Hospital were identified manually using operation theatre notes and the patients’ demographic profiles, comorbidities, laboratory investigation results and treatment methods were extracted from the patients’ files. For patients with multiple admissions for the same diabetic foot problem, laboratory investigation results from the first admission were obtained. Patients with incomplete data were excluded from this study.

In our clinical setting, major amputation was defined as transtibial and transfemoral amputations. There was no transpelvic amputation, hip disarticulation or through-knee amputation performed during the study period. All decisions for lower limb major amputations were made by well-trained orthopaedic surgeons with minimum of seven years orthopaedic training (range: seven to ten years).

The following aspects were assessed to determine the predictive factors of major lower limb amputation; gender, age, source of referral, presence of hypertension, duration of illness prior to presentation, fever, blood glucose level, multiple surgeries, cultures, hemoglobin level, total white blood cell count, urea level and estimated glomerular filtration rate (eGFR). Fever was defined as body temperature of 38.3°C and above. A patient with more than three surgical procedures for the same diabetic foot ulcer was considered having history of multiple surgeries. Cultures were obtained from the first intra-operative tissue samples to avoid contamination and they are classified into no growth, mono-microbial and poly-microbial. All blood investigations were taken upon presentation with the eGFR calculated using the Modification of Diet in Renal Disease (MDRD) equations^13^. Types of diabetic foot complications were also studied to determine any correlation with major amputation of lower limb.

Statistical analysis was performed using STATA version 12.0 with statistical significance set at p < 0.05. Predictor factors of major lower limb amputation were determined using simple logistic regression and multiple logistic regression analysis (Forward LR). Upon simple logistic regression, independent factors with p<0.05 were selected for multiple logistic regression analysis (Forward LR). Multicollinearity and interaction were checked and were not found, indicating each variable was independent. The Hosmer-Lemeshow test, (*p*=0.996), Pearson chi-square test, (*p*=0.954), classification table (overall classified percentage=92.9%) and area under the ROC curve (95.7%) were applied to check the model fitness.

## Results

A total of 170 patients were included in this study. There were 87 males and 83 females with a male to female ratio of approximately 1:1 (Table I). Majority of the patients were referred from Kuala Lipis Hospital (n=92, 54.1%), followed by Raub Hospital (n=44, 25.9%) and Bentong Hospital (n=34, 20.0%) (Table I). The most common form of diabetic foot complication was the infected wound (40.0%). Other types of complications include abscess (24.1%), gangrene (16.5%), necrotising fasciitis (15.3%) and cellulitis (4.7%). Of those 170 patients included in this study, 21 patients (12.4%) underwent major lower limb amputations (15 transtibial and 6 transfemoral).

**Table I:** Descriptive statistics for potential risk factors for major outcome surgery

Risk Factors	Minor Surgery n (%)	Major Amputation n (%)	Total n (%)
Duration of disease (days)^a^	11.3 (14.4)	19.5 (13.8)	12.3 (14.5)
Gender			
Male	79 (90.8)	8 (9.2)	87 (51.2)
Female	70 (84.3)	13 (15.7)	83 (48.8)
Age (Years)			
< 60	100 (93.5)	7 (6.5)	107 (62.9)
≥ 60	49 (77.8)	14 (22.2)	63 (37.1)
Facilities			
Lipis	86 (93.5)	6 (6.5)	92 (54.1)
Bentong	26 (76.5)	8 (23.5)	34 (20.0)
Raub	37 (84.1)	7 (15.9)	44 (25.9)
Hypertension			
No	76 (96.2)	3 (3.8)	79 (46.5)
Yes	73 (80.2)	18 (19.8)	91 (53.5)
Temperature			
< 38.3^o^C	146 (89.6)	17 (10.4)	163 (95.9)
≥ 38.3^o^C	3 (42.9)	4 (57.1)	7 (4.1)
Dxt (10mmol/L)			
< 10	70 (87.5)	10 (12.5)	80 (47.1)
≥ 10	79 (87.8)	11 (12.2)	90 (52.9)
Multiple surgery site			
No	133 (95.7)	6 (4.3)	139 (81.8)
Yes	16 (51.6)	15 (48.4)	31 (18.2)
Culture			
No Growth	69 (94.5)	4 (5.5)	73 (42.9)
Mono	31 (66.0)	16 (34.0)	47 (27.7)
Poly	49 (98.0)	1 (2.0)	50 (29.4)
NF			
No	137 (95.1)	7 (4.9)	144 (84.7)
Yes	12 (46.2)	14 (54.0)	26 (15.3)
Gangrene			
No	124 (87.3)	18 (12.7)	142 (83.5)
Yes	25 (89.3)	3 (10.7)	28 (16.5)
Abscess			
No	108 (83.7)	21 (16.3)	129 (75.9)
Yes	41 (100.0)	0 (0.0)	41 (24.1)
Infected Wound			
No	85 (83.3)	17 (16.7)	102 (60.0)
Yes	64 (94.1)	4 (5.9)	68 (40.0)
Cellulitis			
No	141 (87.0)	21 (13.0)	162 (95.3)
Yes	8 (100.)	0 (0.0)	8 (4.7)
Hb (g/dL)			
≥ 10	101 (98.1)	2 (1.9)	103 (60.6)
< 10	48 (71.6)	19 (28.4)	67 (39.4)
WBC (x109/L).			
< 15	113 (96.6)	4 (3.4)	117 (68.8)
≥ 15	36 (67.9)	17 (32.1)	53 (31.2)
Urea (mmol/L) ^a^	6.7 (4.3)	7.2 (3.2)	6.8 (4.2)
eGFR (ml/min/1.73m^2^)a	74.8 (39.6)	71.5 (29.1)	74.4 (38.4)

Note: ^a^Data presented in mean and standard deviation

Majority of the patients were afebrile upon presentation to the tertiary centre (95.9%) (Table I). Upon presentation to the tertiary centre, more than half (52.9%) of the patients had blood glucose levels of more than 10 mmol/L (Table I).

Approximately one fifth of the patients (18.2%) had undergone more than three surgical procedures for the same diabetic foot wound (Table I). In the aspect of intra-operative tissue cultures, majority of the samples had no growth (42.9%), followed by poly-microbial samples (29.4%) and mono-microbial samples (27.7%).

Upon simple logistic regression analysis, independent factors such as age (p=0.004), source of referral (p=0.011), hypertension (p=0.004), duration of illness (p=0.026), fever (p=0.002), history of multiple surgeries (p<0.001), mono-microbial culture (p=<0.001), necrotising fasciitis (p<0.001), anaemia (p<0.001) and leukocytosis (p<0.001) were found to be associated with major amputation of lower limb (Table II). On the other hand, independent factors such as gender (p=0.205), uncontrolled blood glucose level (p=0.956) and eGRR (p=0.707) were found to be not associated with major amputation of lower limb (Table II). However, upon multiple logistic regression analysis (Forward LR), only longer duration of disease (p=0.002; OR=1.06), raised total white blood cell count (p<0.001; OR=63.2) and history of more than three limb-salvaging surgeries (p<0.001; OR=60.32) were predictors for major amputation of lower limb in diabetic foot infections (Table II).

**Table II:** Associative risk factors of major surgery outcome

Variables	Crude Odds Ratio (95%CI)^a^	P-value	Adjusted Odds Ratio (95%CI)^b^	P-value
Duration of disease (days)^a^	1.03 (1.003, 1.053)	0.026	1.06 (1.023, 1.110)	0.002
Gender				
Male (ref)	1			
Female	1.83 (0.718, 4.680)	0.205		
Age (Years)				
< 60 (ref)	1			
≥ 60	4.08 (1.548, 10.762)	0.004		
Facilities				
Lipis (ref)	1			
Bentong	4.41 (1.402, 13.870)	0.011		
Raub	2.71 (0.853, 8.62)	0.091		
Hypertension				
No (ref)	1			
Yes	6.25 (1.765, 22.105)	0.004		
Temperature				
< 38.3^o^C (ref)	1			
≥ 38.3oC	11.45 (2.301,55.536)	0.002		
Dxt (mmol/L)				
< 10 (ref)	1			
≥ 10	0.97 (0.390, 2.433)	0.956		
Multiple surgery site				
No (ref)	1			
Yes	20.78 (7.061, 61.163)	<0.001	60. 32 (9.202, 395. 326)	<0.001
Culture				
No Growth (ref)	1			
Monomicrobial	8.90 (2.750, 28.822)	<0.001		
Poly microbial	0.352 (0.038, 3.246)	0.357		
Necrotising fasciitis				
No (ref)	1			
Yes	22.83 (7.730, 67.390)	<0.001		
Gangrene				
No (ref)	1			
Yes	0.827 (0.226, 3.020)	0.773		
Abscess				
No (ref)	1			
Yes	Nil	Nil		
Infected Wound				
No (ref)	1			
Yes	0.31 (0.100, 0.973)	0.045		
Cellulitis				
No (ref)	1			
Yes	Nil	Nil		
Hb (g/dL)				
≥ 10 (ref)	1			
< 10	19.99 (4.474, 89.318)	<0.001		
WBC (x109/L)				
< 15 (ref)	1			
≥ 15	13.34 (4.216, 42.214)	<0.001	63.2 (7.874, 507.393)	<0.001
Urea (mmol/L) ^a^	1.03 (0.930,1.137)	0.550		
eGFR (ml/min/1.73m^2^)^a^	1.00 (0.985,1.010)	0.707		

Notes: ^a^Simple Logistic Regression was applied.^b^Forward LR Multiple Logistic Regression was applied. Multicollinearity and interaction were checked and not found. Hosmer-Lemeshow test, (p =0.996), Pearson chi-square test, (p = 0.954), classification table (overall classified percentage =92.9%) and area under the ROC curve (95.7%) were applied to check the model fitness. ref = reference group, Nil = no results available as one of the cell has expected cell count less than 5

## Discussion

Majority of the patients were afebrile upon presentation to the tertiary centre (95.9%) (Table I). This might be due to the initiation of empirical antibiotics and antipyretics at their respective district hospitals prior to referrals. Despite most of the patients had already been started on antidiabetic medications (oral hypoglycemic agents and/or insulin), their blood sugar levels remained uncontrolled upon presentation to the tertiary centre, with more than half (52.9%) of the patients having blood glucose levels of more than 10 mmol/L (Table I).

Approximately one fifth of the patients had undergone more than 3 surgical procedures for the same diabetic foot wound. In the aspect of intra-operative tissue cultures, majority of the samples had no growth (42.9%), followed by poly-microbial samples (29.4%) and mono-microbial samples (27.7%). This may be due to the fact that the tissue samples were obtained after the initiation of empirical antibiotics. In terms of blood investigations, more than two-third of the patients had anemia and raised infective marker (total white blood cell count), possibly due to prolonged infection prior to admission.

Fourteen patients (22.2%) with the age of more than 60 years underwent major amputation of lower extremities as compared to seven patients (6.5%) who were younger than the age 60 years. Patient’s age of more than 60 years was found to be a significant independent risk factor for major amputation (p=0.004). This finding was similar to other studies^14-16^.

A study by Hamalainen *et al* showed that males had a higher risk of major amputation of lower limb^17^. In our study, gender was not found to be a predictive factor for major amputation in diabetic patients (p=0.205). This was comparable to findings by Yusof *et al* and Namgoong *et al*^10, 18^.

In this cluster of three district hospitals, Kuala Lipis Hospital was used as a reference for comparison with Bentong Hospital and Raub Hospital. This was because Kuala Lipis Hospital is a centre with multidisciplinary specialties and patients from Kuala Lipis Hospital were under the care of both medical and orthopaedic specialists, a service which was not available at the other two district hospitals. Treating patients with diabetic foot complications via a multidisciplinary approach showed a better outcome^19^. Patients from Bentong Hospital were associated with a higher risk of major lower limb amputation (p=0.011) whereas there was no such significant association found in patients from Raub Hospital (p=0.091). This may be explained by the close proximity between Raub Hospital and Kuala Lipis Hospital, making earlier referral easier and more convenient logistically.

A meta-analysis by Shin *et al* showed that the presence of hypertension was an independent risk factor for major limb amputation in diabetic foot ulcer, a finding which our study concurs with (p=0.004)^20^. An optimal blood pressure control can slow the progression of end-organ damage in diabetic patients^20^.

The duration of illness was found to be an important predictive factor for major limb amputation (p=0.026). In rural areas, patients with diabetic foot complications tend to present late to the hospitals due to transport difficulties. Late presentation after the onset of diabetic foot complication and subsequently delayed treatment and referral were associated with increased risk of amputation and mortality^21, 22^.

Sepsis accounted for up to three quarter (74%) of incidence in hospitalised patients with fever^23^. Upon presentation to the tertiary centre, most of the patients were afebrile (95.9%). This may be due to the fact that empirical antibiotics and antipyretics have been initiated prior to referrals. Among those patients with fever, more than half of them (57.1%) subsequently underwent major amputation (Table I). Hence, fever was deemed to be an independent risk factor for major amputation of the lower limb (p=0.002). This was consistent with findings by Yusof *et al*, although Aziz *et al* showed otherwise^10, 14^. This could be attributed by a difference in definition of fever used in the studies. Patients with temperature of 38^o^C or higher were considered febrile by Aziz *et al*, whereas we considered a core temperature of 38.3^o^C or higher as fever, based on the recommendation of the American College of Critical Care Medicine, the International Statistical Classification of Diseases, and the Infectious Diseases Society of America^24^.

A good glycemic control was found to be of utmost important to prevent major amputation in some studies^25, 26^. This finding was not replicated in other studies which find that glycemic control is not a significant predictive factor for major amputation^10, 14^. In our local setting, glycosylated haemoglobin was not routinely measured upon admission due to limited resources at district hospitals. By using random blood sugar level as estimation, it was found to be insignificant as a predictive factor for major amputation.

Multiple limb-salvaging surgeries were often performed for diabetic foot infections although some of the patients may end-up with major amputations^27^. We found that almost half of the patients (48.4%) who have undergone more than three times limb-salvaging surgeries for diabetic foot infections end up with major amputations. Hence, history of more than three times of limb-salvaging surgeries was a predictive factor in major amputation of diabetic foot infections (p<0.001).

In terms of causative pathogens, mono-microbial culture was noted to be a predictive factor for major amputation (p<0.001). This was similar to the finding by Aziz *et al*^14^. It should be noted that all the intra-operative tissue samples were collected after the initiation of empirical antibiotics, either by treating physicians at district hospitals or upon presentation to the emergency department at the referral centre. This may explain the reason of a high percentage of tissue samples which had no pathogen growth (42.9%) (Table I). We included only intra-operative tissue cultures as swab cultures were associated with higher risk of missed pathogens, especially Gram-negative bacteria^28^.

We classified diabetic foot complications into five broad categories, namely necrotising fasciitis, gangrene, abscess, infected wound and cellulitis. The diagnosis was further confirmed during intra-operative examination. None of the patients with abscess or cellulitis underwent major amputation. Necrotising fasciitis was a predictive factor for lower limb major amputations in diabetic patients (p<0.001) whereas gangrene was found to be on the contrary (p=0.773). Interestingly, patients with infected wounds were noted to be less likely to undergo major amputations (odd ratio=0.31, p=0.045).

Various studies had demonstrated low haemoglobin level to be a predictive factor for major amputation and mortality^10, 14, 29, 30^. Haemoglobin level of less than 10 g/dL was a significant predictive factor for major amputation (p<0.001) in our study. About 28.4% of patients with haemoglobin level less than 10 g/dL underwent major amputations of lower limbs (Table I). This was because diabetic patients with lower circulating haemoglobin will have reduced oxygen delivery to the affected tissues, subsequently leading to impairment of compensatory response during infections^30^.

Infective marker in the form of total white blood cell count had been consistently shown to be a predictive factor for major amputation in diabetic patients^10, 14, 29^. Leukocytosis, a sign of inflammation, was a predictive factor for major amputation of lower limb (p<0.001) with almost one third of our patients (32.1%) with leukocytosis upon presentation will progress to major amputation of their lower limbs.

One of the microvascular complications of diabetes mellitus was nephropathy^31^. Margolis *et al* showed that patients with chronic kidney disease (CKD) were more prone to develop diabetic foot ulcers^31^. However, contradicting results were seen in several studies which explore the association between renal function and major amputation. Some of the researches showed that impaired renal function was a predictive factor for major amputation while others found no association between renal function and amputation in diabetic patients^10, 14, 31^. In our study, there was no correlation between urea level (p=0.550) or eGFR (p=0.707) and major amputation in diabetic foot complications.

Our study had several limitations. First of all, most of our patients received either oral or intravenous antibiotic(s) at the health clinics or district hospitals prior to their presentations to the tertiary centre, therefore bacterial cultures obtained may not be representative of the true incidence of the causative pathogens in diabetic foot infections. Other studies found that peripheral vascular disease and albumin level were among the determinant factors for major amputation^10, 14^. However, these factors were not included in this study. This study only involved patients operated in Kuala Lipis Hospital with majority of the cases referred from Raub Hospital and Bentong Hospital, hence the predictive factors are only applicable to these district hospitals. Nonetheless, this is the first study which primarily focuses on the district setting with limited resources, serving as a guide for other study to be conducted in other districts.

## Conclusion

Among the factors analysed in this study, a longer duration of disease, raised total white blood cell count and history of more than three limb-salvaging surgeries were identified as predictors for major amputation of lower limb in diabetic foot infections using stepwise logistic regression analysis. Thus, we recommend treating physicians at district hospitals for early referral and aggressive management of diabetic patients with those risky conditions.
